# Acute porphyrias – A neurological perspective

**DOI:** 10.1002/brb3.2389

**Published:** 2021-10-17

**Authors:** Lea M. Gerischer, Franziska Scheibe, Astrid Nümann, Martin Köhnlein, Ulrich Stölzel, Andreas Meisel

**Affiliations:** ^1^ Charité – Universitätsmedizin Berlin, corporate member of Freie Universität Berlin and Humboldt‐Universität zu Berlin, Department of Neurology Berlin Germany; ^2^ Charité – Universitätsmedizin Berlin, corporate member of Freie Universität Berlin and Humboldt‐Universität zu Berlin, NeuroCure Clinical Research Center Berlin Germany; ^3^ Department of Internal Medicine II, Porphyria Center Saxonia Klinikum Chemnitz gGmbH Chemnitz Germany

**Keywords:** acute porphyria, autoimmune encephalitis, Guillain‐Barré syndrome, porphyric encephalopathy, porphyric neuropathy

## Abstract

Acute hepatic porphyrias (AHP) can cause severe neurological symptoms involving the central, autonomic, and peripheral nervous system. Due to their relative rarity and their chameleon‐like presentation, delayed diagnosis and misdiagnosis are common. AHPs are genetically inherited disorders that result from heme biosynthesis enzyme deficiencies and comprise four forms: acute intermittent porphyria (AIP), variegate porphyria (VP), hereditary coproporphyria (HCP), and ALA‐dehydratase porphyria (ALADP). Depending on the clinical presentation, the main differential diagnoses are Guillain‐Barré syndrome and autoimmune encephalitis. Red flags that could raise the suspicion of acute porphyria are neurological symptoms starting after severe (abdominal) pain, in association with reddish urine, hyponatremia or photodermatitis, and the presence of encephalopathy and/or axonal neuropathy. We highlight the diagnostic difficulties by presenting three cases from our neurological intensive care unit and give a comprehensive overview about the diagnostic findings in imaging, electrophysiology, and neuropathology.

## Graphical abstract

Acute hepatic porphyrias (AHP) can cause severe neurological symptoms involving the central, autonomic and peripheral nervous system. Due to their relative rarity and their chameleon‐like presentation awareness among neurologists is low and delayed diagnosis and misdiagnosis are common. Red flags that should raise the suspicion of acute porphyria are neurological symptoms starting after severe (abdominal) pain, in association with reddish urine, hyponatremia or photodermatitis and the presence of encephalopathy and/or axonal neuropathy.

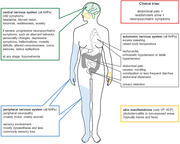

## 1 INTRODUCTION

The porphyrias comprise a group of genetically inherited disorders that result from enzyme deficiencies within the pathway of heme biosynthesis (Karim et al., 2015; Puy et al., 2010). Neurologists should be aware of four of the eight forms that can cause acute neurological symptoms: acute intermittent porphyria (AIP), variegate porphyria (VP), and hereditary coproporphyria (HCP) are autosomal dominant. The extremely rare form of ALA‐dehydratase porphyria (ALADP) is autosomal recessive and only causes symptoms in the presence of biallelic pathogenic variants (i.e., homozygous or compound heterozygous variants). The enzyme deficiencies in these four lead to metabolic dysfunction and overproduction of porphyrin precursors in the liver. They are summarized as acute hepatic porphyrias (AHP). The other forms mainly induce cutaneous symptoms and are summarized as photocutaneous porphyrias (Karim et al., 2015; Puy et al., 2010). In this article, we will focus exclusively on the four AHPs that cause acute neurological symptoms.

**FIGURE 1:**
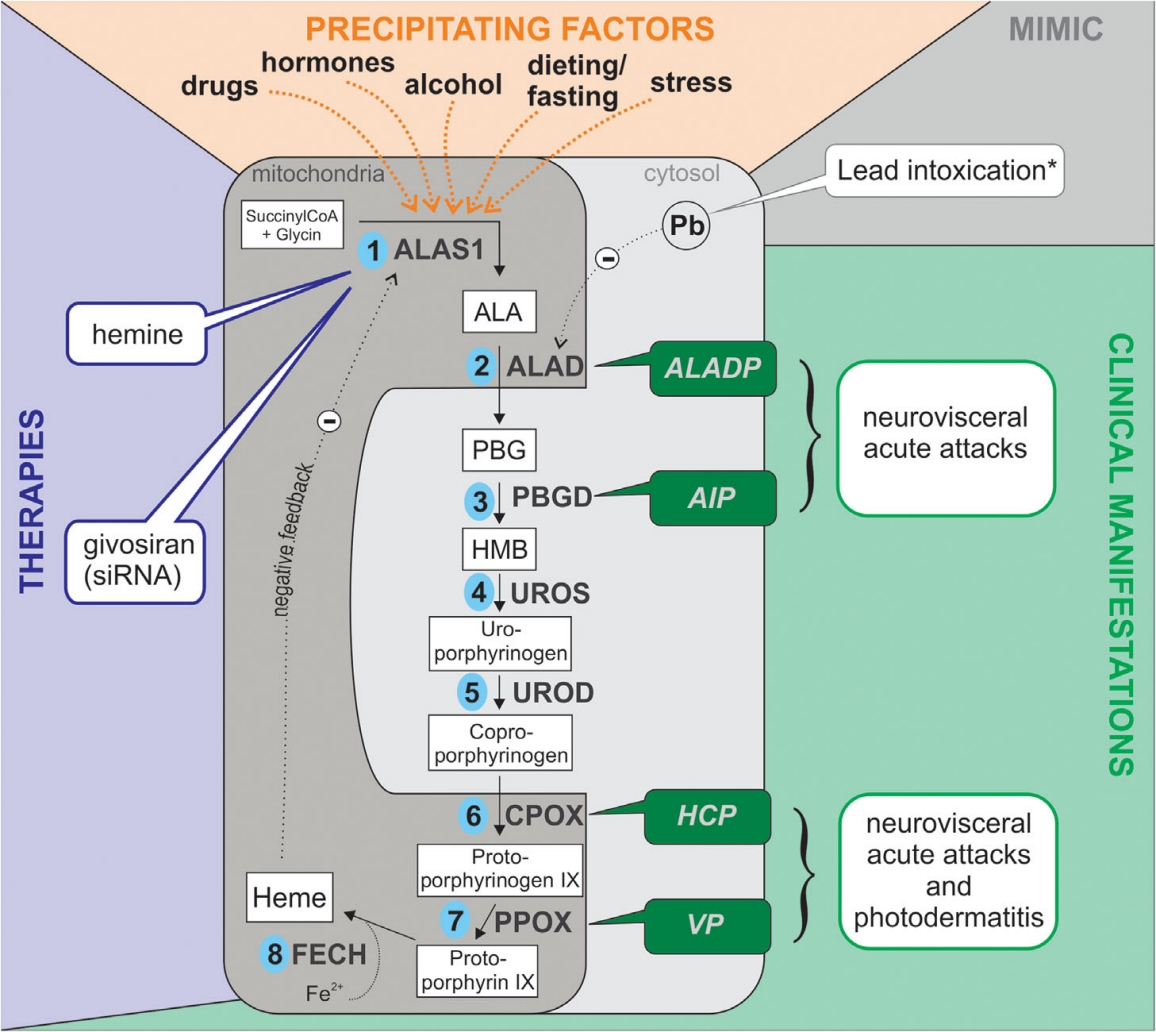
Heme biosynthesis pathway and the four acute hepatic porphyrias, clinical manifestations, precipitating factors and therapies. In the liver, heme regulates the first enzyme ALAS1 via a negative feedback loop. In contrast to the liver enzyme ALAS1, the rate‐limiting enzyme ALAS2 in the bone marrow is regulated by iron and erythropoietin instead of heme. *A lead intoxication can mimic the clinical and biochemical constellation found in ALADP and should therefore always be excluded. AIP, acute intermittent porphyria; ALA, 5‐aminolaevulinic acid; ALAD, ALA‐dehydratase; ALADP, ALA‐dehydratase porphyria; ALAS, ALA‐synthase; CPOX, coproporphyrinogen oxidase; Fe2+, ferrous iron; FECH, ferrochelatase; HCP, hereditary coproporphyria; HMB, hydroxymethylbilane; Pb, lead; PBG, porphobilinogen; PBGD, porphobilinogen deaminase; PPOX, protoporphyrinogen oxidase; UROD, uroporphyrinogen decarboxylase; UROS, uroporphyrinogen III synthase; siRNA, small interfering RNA; SuccinylCoA, succinyl‐coenzyme‐A; VP, variegate porphyria

An overview of the heme biosynthesis pathway is given in Figure 1. The enzyme deficiency is specific for each disorder. Precipitating factors that upregulate heme biosynthesis cause an accumulation of the metabolite upstream from the deficient enzyme. The porphyrin precursors aminolevulinic acid (ALA) and possibly also porphobilinogen (PBG) are considered neurotoxic. ALA, PBG, and porphyrins can be detected during an acute attack in urine, feces or blood depending on their water solubility (Anderson et al., 2005; Karim et al., 2015; Puy et al., 2010; Simon & Herkes, 2011; Stein et al., 2017). Table 1 gives an overview of the laboratory findings.

**TABLE 1:** Overview of the acute porphyrias: Deficient enzyme, genetics, laboratory findings and clinical presentation

					Laboratory findings				
Form of porphyria	Inheritance	Gene	Gene locus	Deficient enzyme; activity % of normal	Urine	Feces	Plasma (fluorescence emission peak in nm^c^)	Clinical presentation	Comment
ALADP	AR	ALAD	9q34	ALA dehydratase; ∼5%	ALA ++ Uroporphyrin + Coproporphyrin ++	—	—	Acute neurovisceral attacks	Rare; exclude lead poisoning
AIP	AD	HMBS^b^	11q23.3	Porphobilinogen deaminase; ∼50%	ALA, PBG ++ Uroporphyrin + Coproporphyrin +/‐	Coproporphyrin +/− Protoporphyrin +/−	615‐620	Acute neurovisceral attacks	Erythrocyte PBGD (isoenzyme) usually decreased
HCP	AD	CPOX	3q12	Coproporphyrinogen oxidase; ∼50%	ALA, PBG + Uroporphyrin +/‐ Coproporphyrin +	Coproporphyrin III + Protoporphyrin +/−	615‐620	Acute neurovisceral attacks AND photosensitivity	CPOX activity in leucocytes^a^
VP	AD	PPOX	1q22	Protoporphyrinogen oxidase; ∼50%	ALA, PBG + Uroporphyrin + Coproporphyrin +	Coproporphyrin III + Protoporphyrin +	624‐627	Acute neurovisceral attacks AND photosensitivity	PPOX activity in leucocytes^a^; Plasma fluorescence emission peak also in remission

*Note*: Laboratory findings: ++ markedly raised; + raised; +/− may be raised or normal.

*Abbreviations*: AD, autosomal dominant; AIP, acute intermittend porphyria; ALA, 5‐aminolaevulinic acid; ALAD, ALA dehydratase; ALADP, ALA dehydratase porphyria; AR, autosomal recessive; CPOX, Coproporphyrinogen oxidase; HCP, hereditary coproporphyria; HMBS, hydroxymethylbilane‐synthase; nm, nanometers; PBG, porphobilinogen; PPOX, protoporphyrinogen oxidase; VP, variegate porphyria.

^a^ Only available in some specialized laboratories.

^b^ Formerly known as PBGD (porphobilinogen deaminase).

^c^ Excitatory wave length 405 nm.

The clinical presentation varies greatly and AHPs can mimic many other (often more common) neurological or psychiatric diseases. In 1937, Waldenström described acute porphyria as the “little imitator,” contrasting it with syphilis, the “big imitator” of the early 20th century (Crimlisk, 1997; Waldenstrom, 1957). The typical clinical pattern consists of acute abdominal pain accompanied by signs of autonomic dysfunction followed by neuropsychiatric disturbances with signs of encephalopathy and development of peripheral neuropathy. Dark or reddish urine may prompt the clinician's suspicion, but does not always occur (Pischik & Kauppinen, 2009). In AIP, only neurovisceral attacks occur, whereas VP and HCP can cause both neurovisceral attacks and cutaneous symptoms. However, the clinical pattern alone does not allow distinction between the four AHPs and laboratory workup is required for precise diagnosis.

Women are more often affected than men. For AIP, the prevalence of probable pathogenic mutations is about 560 per million (Chen et al., 2016). However, the prevalence of overt AIP is only estimated at 5.4 per million in Europe, (Elder et al., 2013) as approximately 60%–90% of gene mutation carriers remain asymptomatic (Stein et al., 2017). Reliable estimations are difficult due to frequent misdiagnosis or delayed diagnosis (Anderson et al., 2005; Puy et al., 2010; Tracy & Dyck, 2014).

The aim of this article is to raise awareness for AHPs among neurologists. We highlight the diagnostic difficulties by reporting three cases (overview in table 2) from our neurological intensive care unit (NICU) and give a comprehensive overview of the neurological manifestations, diagnostic findings, and therapeutic strategies.

**TABLE 2:** Summary of the three cases

	Case 1	Case 2	Case 3
Age at presentation, sex	75, female	40, female	29, female
Form of AHP	AIP	VP	AIP
Previous misdiagnosis	GBS	Irritable bowel syndrome/somatoform disorder	Autoimmune encephalitis
Clinical presentation	Porphyric neuropathy	Porphyric neuropathy and mild encephalopathy	Porphyric encephalopathy
Additional signs			
Reddish urine	Present	Present	Not present
Hyponatremia	No	Severe (106 mmol/L)	No
Main neurological symptoms	Dizziness, near‐fainting, diffuse abdominal pain, general weakness, gait instability	Intermittent disorientation, excessive sleepiness, diffuse vision loss, general weakness, and gait instability	Restlessness, insomnia, dizziness, slowed speech, followed by aggressive/ erratic behavior, incoherent speech, and confusional state
Neurological examination	Bilateral facial paresis, dysarthria, accessory nerve involvement, severe flaccid tetraparesis with proximal accentuation, deep tendon reflexes absent	Cognitive slowing, memory deficits, reduced vision, proximally and lower extremity accentuated flaccid tetraparesis, deep tendon reflexes absent, mild distal hypoesthesia	Confusion, severe dysarthria, dysphagia, bradykinetic syndrome, gait ataxia and overall reduced muscle strength (4/5), deep tendon reflexes normal
Neurological workup			
Brain imaging	Normal	Normal	Brain MRI with T2‐hyperintensities bilaterally in the basal ganglia without contrast enhancement
EEG	—	—	Diffuse slowing of activity
CSF	Albuminocytological dissociation with protein level 570 mg/L (nor 150–450 mg/L)	Albuminocytological dissociation with protein level 625 mg/L (norm 150–450 mg/L)	Normal
NCS	Mainly motor, axonal neuropathy, mild signs of demyelination, mild sensory involvement	Severe axonal, mainly motor neuropathy with lower limb accentuation	No signs of neuropathy
Neuropathology	Sural nerve biopsy: reduction of myelinated fibers, signs of ongoing axonal damage; muscle biopsy: massive chronic‐neurogenic damage	—	—
Outcome	Death	Persistent foot dorsiflexor paresis	Complete recovery

*Abbreviations*: “−,“ not done; AHP, acute hepatic porphyria; AIP, acute intermittent porphyria; CSF, cerebrospinal fluid; EEG, electroencephalogram; NCS, nerve conduction studies; VP, variegate porphyria.

## 2 CASE PRESENTATIONS

### 2.1 Porphyric neuropathy mimicking GBS—Case 1

A 75‐year‐old female was transferred to our NICU with suspected GBS. Previous medical history was unremarkable except for arterial hypertension. Four weeks prior to admission, the patient had suffered a vertebral fracture (L2) that had been treated with pain killers (tramadol and metamizole) and a corset. Within days after discharge, she had complained of dizziness, near‐fainting, diffuse abdominal pain, and diarrhea. Upon readmittance, she was dehydrated, tachycardic, hypotensive, and displayed orthostatic intolerance. Gastroenteritis was suspected and treated with intravenous fluids and antibiotics. Further investigations revealed no causal infectious pathogen. Within days, the diarrhea stopped. However, she developed episodes of supraventricular tachycardias and complained of general weakness. Twenty‐nine days after symptom onset, mild tetraparesis was noted and the patient was moved under neurological care. Neurological examination revealed a moderate tetraparesis with proximal accentuation and mild bilateral facial paresis. Intravenous immunoglobulin (IVIG) was started and because of the worsening tetraparesis, the patient was transferred to our NICU.

Upon admission, we saw an awake, fully orientated patient with discrete bilateral facial paresis, mild dysarthria, mild accessory nerve involvement, severe flaccid tetraparesis with proximal accentuation, absence of deep tendon reflexes, and lack of sensory complaints. CSF showed slight protein increase of 570 mg/L (norm 150–450 mg/L) without pleocytosis (termed albuminocytological dissociation). Nerve conduction studies (NCS) revealed a mainly motor, axonal polyradiculoneuropathy with mild signs of demyelination (prolonged or absent F‐wave latencies) and mild sensory involvement. Laboratory investigation revealed mild hypocalcemia and hypomagnesemia. Sodium, potassium, creatinine, and blood count were normal. Liver enzymes and CRP were minimally raised.

Despite IVIG over 5 days, the tetraparesis deteriorated and the patient was intubated. CSF and serum investigations revealed a CMV‐reactivation in the serum and ganciclovir was started. All other investigations revealed no infectious cause. Ganglioside and onconeural antibodies were negative.

After 4 weeks in NICU, the patient had meanwhile been tracheotomized and continued to be mechanically ventilated. Since no relevant progress was seen regarding neurological deficits, plasmapheresis was started. After five sessions, facial paresis improved mildly. Nerve‐muscle‐biopsy showed massive chronic‐neurogenic damage in the muscle, reduction of myelinated fibers and signs of ongoing axonal damage in the nerve, and no signs of vasculitis. We started another cycle of plasmapheresis and treated with prednisolone (2 mg/kg body weight) concomitantly.

On day 45 of NICU treatment, red‐brown urine was noted for the first time, leading to suspicion of porphyria and urine was sent for PBG, ALA, and porphyrin analysis. Urine color normalized within 2 weeks. The results showed significantly raised metabolite levels (see Table SI). A suspected diagnosis of acute porphyria was made and the patient was treated with hemin (3 mg/kg body weight) for 5 days. Arm paresis and cognition improved significantly.

AIP‐diagnosis was supported by enzyme analysis (reduced erythrocyte porphobilinogen deaminase [PBGD] activity of 43% [8.1 nmol/s; norm 13.3–24.7 nmol/s]) and confirmed through genetic testing (novel heterozygous mutation in exon 15 of the HMBS‐gene; variant c.966delC). The family and the GP confirmed that this episode was the first manifestation of AIP.

The patient was transferred to rehabilitation after five months. Neurological status before discharge showed an alert patient, capable of performing simple tasks and communicating personal needs, but still requiring mechanical ventilation. Facial paresis and arm strength had improved, but flaccid paraplegia persisted. Four weeks after transfer to the rehabilitation center, the patient developed a sudden coma and tachypnea and died. No autopsy was performed.

### 2.2 Worsening abdominal pain leading to neuropsychiatric symptoms and severe neuropathy—Case 2

A 40‐year‐old female was transferred to our NICU with suspected porphyric neuropathy. Symptoms had started three months prior with recurrent and severe epigastric pain accompanied by nausea and vomiting. Abdominal ultrasound and gastroscopy had been normal and proton‐pump inhibitors had not improved symptoms. A diagnosis of irritable bowel syndrome had been proposed. The pain had worsened and led to reduced appetite, weight loss and general weakness, and she was admitted to an external hospital. Initial laboratory results were unremarkable, but the patient was intermittently disorientated, showed excessive sleepiness and continued to complain of severe pain despite painkillers. Later, the patient developed severe hyponatremia and reported dark reddish urine. The combination of unexplained abdominal pain, dark urine, and hyponatremia led to suspicion of acute porphyria and glucose infusions were started. Because of worsening hyponatremia (106 mmol/L), she was transferred to our center.

Upon transfer, the patient was alert and orientated, but reported diffuse vision loss, general weakness and gait instability. Neurological examination revealed pronounced cognitive slowing, memory and concentration deficits, reduced vision, dysmetrical eye bulb movements, a proximally and lower extremity accentuated flaccid tetraparesis with absent deep tendon reflexes, and mild distal hypoesthesia with sock‐like distribution. Laboratory results confirmed severe hyponatremia (106 mmol/L), hypokalemia (2.4 mmol/L), and hypocalcemia. Creatinine levels, liver enzymes, and blood count were normal. CSF results showed an albuminocytological dissociation with protein level of 625 mg/L (norm 150–450 mg/L) and were otherwise unremarkable. NCS showed a severe axonal, mainly motor neuropathy with lower limb accentuation. Considering the clinical picture with abdominal pain, severe hyponatremia, mild signs of encephalopathy, and severe neuropathy, acute porphyria was the main suspected diagnosis and urine analysis was ordered. PBG levels were significantly raised at 132 mg/L (norm < 1.7 mg/L). Hemin was started and glucose infusions, and sodium and potassium supplementation continued. However, despite sodium levels slowly rising (125 mmol/L), the patient's condition deteriorated further with hallucinations and progressive tetraparesis, and she had to be intubated. We continued hemin for 5 days. By this time, sodium levels were normal again. Results of urine and stool analyses are shown in Table SII. Diagnosis of VP was confirmed by positive plasma‐fluorescence‐scan (emission wave length of 627 nm) (Hift et al., 2004) and through genetic testing (known heterozygous mutation in exon 6 of the PPOX‐gene; mutation c.503G > A, p.Arg168His) (Frank et al., 1998; Makki et al., 2017).

After reduction of analgosedation, the patient was alert, capable of communicating by head and eye movements and using an eye‐tracking software. Due to persistent flaccid tetraparesis, tracheotomy was performed. MRI of head and spine were normal. Tetraparesis started to improve slightly after 3 weeks with distal strength improving first. Severe burning abdominal and lower extremity pain was treated with morphine, pregabalin, and duloxetine.

After 22 days in NICU, the patient was transferred to rehabilitation. Neurological examination showed improvement of tetraparesis, absent deep tendon reflexes, and persisting allodynia and hypoesthesia in the legs. The patient had started weaning from the respirator. The patient recovered over the course of one year but retained bilateral foot dorsiflexor paresis. Three years later, she suffered from another acute porphyric attack manifesting with intense abdominal pain only. She was treated with glucose infusions and did not develop any new neurological deficits. Laboratory results of remission period and second attack are shown in Table SII.

### 2.3 Porphyric encephalopathy mimicking autoimmune encephalitis—Case 3

A 29‐year‐old female was repatriated to our NICU with suspected NMDA‐receptor antibody encephalitis. On a business trip to a foreign country, she had developed restlessness, insomnia, dizziness, and slowed speech. This had progressed within days to aggressive/erratic behavior and a confusional state with incoherent speech. She was brought to an emergency room and admitted neurologically. Initial laboratory investigations, brain CT and MRI and CSF results were unremarkable. Investigations for infectious causes were negative. A second MRI revealed T2‐hyperintensities bilaterally in the basal ganglia without contrast enhancement (Figure SI). Autoimmune encephalitis was suspected and high‐dose steroids were started. Because of two episodes with loss of consciousness, seizures were suspected and levetiracetam was started to prevent seizures before transfer to our NICU was organized.

Upon admission to our ward, the patient was awake, but confused. Neurological examination revealed severe dysarthria, dysphagia, and a bradykinetic syndrome. She was unable to stand or walk, muscle strength was generally slightly reduced, deep tendon reflexes normal. Blood pressure was within the normal range. We continued high‐dose steroids (methylprednisolone 1 g intravenously for 5 days) and repeated the lumbar puncture. Again, basic CSF results were unremarkable. EEG showed diffuse slowing of activity. MRI findings confirmed symmetrical T2 and DWI hyperintensities bilaterally in the basal ganglia without contrast enhancement compatible with a hepatic/metabolic encephalopathy (Figure SI). However, there were no hepatic or metabolic abnormalities including ammonia.

The patient continued to suffer from anxiety attacks and displayed pronounced memory deficits. She received a beta‐blocker for tachycardia and benzodiazepines for agitation. Because of worsening cognitive deficits, we initiated plasmapheresis. Detailed history with family members revealed that 1 week prior to developing the confusional state, the patient's third molars had been removed. Shortly after, she had developed a painful cheek swelling and taken amoxicillin. The next day, she had developed nausea, vomiting, and cramping abdominal pains followed by predominantly lower back and thigh pain. She was presented to an emergency room where spot urine testing and kidney ultrasound were unremarkable. She was prescribed painkillers and discharged.

Because of the history of abdominal pain followed by an encephalopathic syndrome, urine was sent for porphyrin analysis (PBG, ALA, porphyrins). Meanwhile, results from antibody investigations including NMDA‐receptor antibodies and all investigations for infectious causes returned negative. After 2 days of plasmapheresis, cognition and dysarthria had slightly improved.

Porphyrin analysis showed significantly raised precursors (PBG, ALA) and porphyrins (Table SIII). Therefore, we suspected an acute porphyria and sent blood, urine, and stool for further analyses and excluded a lead poisoning. We immediately stopped all possibly unsafe medications, discontinued plasmapheresis, and started hemin and glucose infusions. The patient improved so rapidly, she was able to eat and get out of bed within 2 days. She continued to improve under hemin for 5 days and was transferred to the normal neurological ward. Neurological examination revealed remaining deficits of hand dexterity, slightly bradykinetic movements, slight dysarthria, and mild gait instability. She had recovered to full strength in arms and legs, dysphagia had normalized. She remained hypomanic and sleepless. NCS showed no signs of neuropathy.

AIP‐diagnosis was confirmed through reduced erythrocyte PBGD‐activity of 48% (9.2 nmol/s; norm 13.3–24.7 nmol/s) and genetic testing (heterozygous mutation in exon 2 of the HMBS‐gene; mutation c.53delT, p.Met18Argfs*3). One‐year follow‐up showed complete clinical recovery. She had not suffered from any further attacks, but showed persistently raised precursors and porphyrins which remained lower than during the attack (Table SIII).

## 3 WHEN TO CONSIDER ACUTE PORPHYRIAS IN NEUROLOGICAL PATIENTS

In recent years, autoimmune encephalitis—despite being a rare disease—has evolved to an important differential diagnosis in unclear neurological cases. If syphilis was the “big imitator” of early 20th century neurology, then autoimmune encephalitis seems to replace it today. However, if a diagnosis becomes a “fashion,” the risk of overlooking other diseases increases. Bearing this in mind, we aim to raise awareness among neurologists for AHPs and their chameleon‐like neurological manifestations. Most importantly, AHP are treatable conditions and disability can be prevented if diagnosed and treated in the early stage. In our opinion, AHPs should be considered routinely as differential diagnosis in cases with (axonal) GBS, suspected autoimmune encephalitis or encephalopathy, and when there are combined signs of central and peripheral nervous system involvement. Clinicians should not rely on the “classical” presentation with abdominal pain and reddish urine, as these signs may be transient or completely absent during an acute attack.

The presented cases highlight the different clinical patterns that should prompt neurologists to exclude AHPs. Cases 1 and 2 highlight the diagnostic challenges in differentiating porphyric neuropathy from GBS. Red flags in both cases were the proximal accentuation of pareses. Case 1 had multiple signs of autonomic dysfunction prior to developing neurological symptoms, whereas in GBS, autonomic dysfunction usually develops parallel to the paresis. In case 1 however, GBS seemed probable as post‐infectious complication after gastrointestinal infection or in association with CMV‐reactivation. Both are known triggers for developing a GBS (van den Berg et al., 2014). Furthermore, in both cases, albuminocytological dissociation in CSF seemed to support GBS‐diagnosis. However, this is a common finding in AHPs as well (Windebank & Bonkovsky, 2005).

Case 3 demonstrates that porphyria can mimic the encephalopathic syndrome seen in autoimmune encephalitis. Abdominal pain was not a leading symptom and had existed for only a few days, followed by back and thigh pain. This sequence was only discovered through detailed history taking, a basic skill for which, unfortunately, time is often scarce in modern medicine, especially in the ICU setting.

As a further consideration, the patient in case 2 had a previous history of recurrent abdominal pain. Since nothing “somatic” had been revealed in multiple investigations, an irritable bowel syndrome or somatoform disorder had been suspected. Many AHP patients receive psychosomatic diagnoses because they are told, that “everything” that causes somatic pain has been excluded (Anderson et al., 2005).

### 3.1 Variability of clinical symptoms during acute attacks

Most acute attacks begin with severe **abdominal pain**, which can be constant or colicky, generalized or localized, and can radiate to the back or thighs (Gouya et al., 2020; Kauppinen, 2005; Puy et al., 2010). The pain is often accompanied by nausea, vomiting, constipation or diarrhea, and abdominal distension and might prompt an exploratory laparotomy (Anderson et al., 2005; Bonkovsky et al., 2014). Signs of **autonomic dysfunction** include resting tachycardia, orthostatic hypotension, systolic and/or labile hypertension, excess sweating, urinary retention, and raised body temperature (Puy et al., 2010). Autonomic function testing during attacks can confirm severe autonomic dysfunction (Laiwah et al., 1985) and striking abnormalities of gastrointestinal motility (Berlin & Cotton, 1950). It has been hypothesized that the abdominal pain itself is caused by autonomic neuropathy (Meyer et al., 1998; Pischik & Kauppinen, 2009) through gastroparesis and disturbed intestinal motility (Berlin & Cotton, 1950) or partly due to intestinal ischemia caused by vasospasms (Lithner, 2000).

Mild **neuropsychiatric symptoms** such as insomnia, restlessness, agitation, and anxiety may be prodromal signs of an incipient acute attack or may develop simultaneously. During severe attacks, this can progress to aberrant behavior, personality changes, depressive symptoms and hallucinations (Anderson et al., 2005; Gouya et al., 2020; Pischik & Kauppinen, 2009).

Signs of **central nervous system involvement** may begin with headache and blurred vision and can progress to vision loss and signs of diffuse encephalopathy with altered consciousness and coma. Seizures and status epilepticus (Dawit et al., 2019; Engelhardt et al., 2004; Zaatreh, 2005) have been described as part of severe acute attacks and have often been attributed to severe hyponatremia or hypomagnesemia (Puy et al., 2010). However, cases with seizures with normal electrolyte levels have also been described and therefore vasogenic edema leading to PRES‐like changes has been suggested as an alternative pathophysiological mechanism (Pischik & Kauppinen, 2009). Severe **hyponatremia** can occur and is interpreted as hypothalamic involvement and inappropriate antidiuretic hormone secretion (Anderson et al., 2005). Others have discussed an association with excess gastrointestinal or renal sodium loss, intravenous fluids (especially glucose infusions), or a combination of these factors (Anderson et al., 2005; Sack, 1990; Stein et al., 2017).

**Peripheral neuropathy** usually develops last during an attack (after abdominal pain and central nervous involvement), on average, within one month after symptom‐onset and is mainly motor and mainly axonal (Albers & Fink, 2004; Simon & Herkes, 2011). NCS may also reveal signs of demyelination (Pischik & Kauppinen, 2009). Onset can be symmetrical or asymmetrical and suspicion of porphyric neuropathy may be prompted by weakness beginning in the arms or proximally in the legs (Albers & Fink, 2004; Pischik & Kauppinen, 2009). In severe cases, this may progress to quadriplegia and respiratory insufficiency, requiring mechanical ventilation (Albers & Fink, 2004; Anderson et al., 2005; Simon & Herkes, 2011). Sensory involvement is usually less severe and may have a stocking‐glove, a proximal bathing‐suit or a patchy and variable distribution, sometimes leading to suspicion of a conversion disorder (Crimlisk, 1997; Pischik & Kauppinen, 2009; Simon & Herkes, 2011). Cranial nerve involvement (IIIrd, VIIth, Xth nerve) has been reported in up to 75% of patients with porphyric neuropathy (Barraza et al., 2012; Pischik & Kauppinen, 2009; Simon & Herkes, 2011).

**FIGURE 2:**
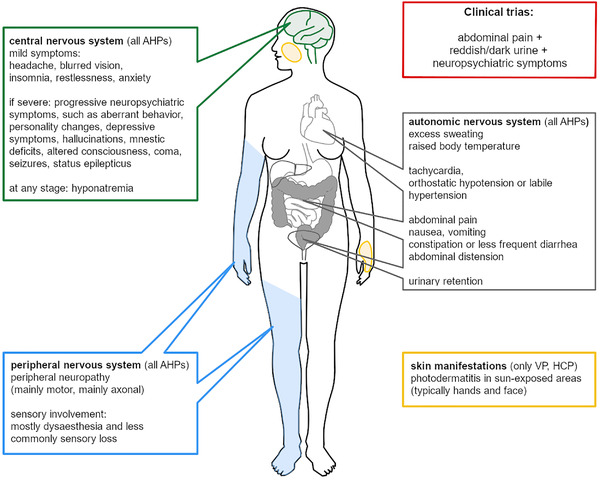
Overview of the clinical neurological manifestations of acute hepatic porphyrias. AHPs, acute hepatic porphyrias; HCP, hereditary coproporphyria; VP, variegate porphyria

Further, extensive fatigue, myalgia, and diffuse muscle weakness have been described during acute attacks (Martasek, 1998; Pischik & Kauppinen, 2009). VP and HCP can cause **skin manifestations** that present as classic photodermatitis in sun‐exposed body areas (face, forearms, dorsa of hands and feet, and neck). (Crimlisk, 1997; Kirsch et al., 1998; Martasek, 1998). These may precede acute neurovisceral attacks or occur independently. Figure 2 illustrates the clinical manifestations.

### 3.2 Neurological differential diagnosis

AHPs can affect both the peripheral and the central nervous system. For cases with severe peripheral neuropathy, the most important differential diagnosis is Guillain‐Barré syndrome (GBS), followed by CIDP and poisoning from lead, arsenic, or thallium (Schutte et al., 2015; Tracy & Dyck, 2014). For cases mainly presenting with signs of diffuse encephalopathy, infectious and autoimmune encephalitis, sinus vein thrombosis, and metabolic encephalopathy should be excluded (Pischik & Kauppinen, 2009). Table 3 gives an overview of the neurological differential diagnoses.

**TABLE 3:** Neurological differential diagnosis

Main symptom	Differential diagnoses
Acute porphyric neuropathy (mainly motor, mainly axonal)	
	(Atypical) Guillain‐Barré syndrome (AMAN‐variant)
	CIDP
	Heavy metal toxicity (lead, thallium, arsenic)
	Paraneoplastic syndromes
	Motoneuron diseases
	Severe vitamin deficiency (e.g., Vitamin B1, B6, B12)
	Diabetes mellitus
	Alcohol‐induced neuropathy
	Medication toxicity (e.g., cisplatin, bortezomib, linezolid)
Porphyric encephalopathy	
	Autoimmune encephalitis
	Infectious encephalitis
	Sinus vein thrombosis
	Metabolic encephalopathy (e.g., uremic, hepatic)
	Mitochondriopathies
	Primary CNS vasculitis
Central AND peripheral nervous system involvement	
	Vasculitis (e.g., giant cell arteritis, polyarteritis nodosa, ANCA‐associated arteritis, cryoglobulinemic vasculitis)
	Systemic rheumatological disorders (e.g., SLE, Sjögren)
	Neurosarcoidosis
	Systemic infections (exclude HIV, borreliosis, syphilis)
Special considerations in children and adolescents	
	Tyrosinemia type I
	Rare variants of late‐onset disorders of the homocysteine metabolism and of the urea cycle

*Abbreviations*: AMAN, acute motor axonal neuropathy; CIDP, chronic inflammatory demyelinating polyneuropathy; SLE, systemic lupus erythematosus.

## 4 LABORATORY DIAGNOSIS OF AN ACUTE ATTACK

**FIGURE 3:**
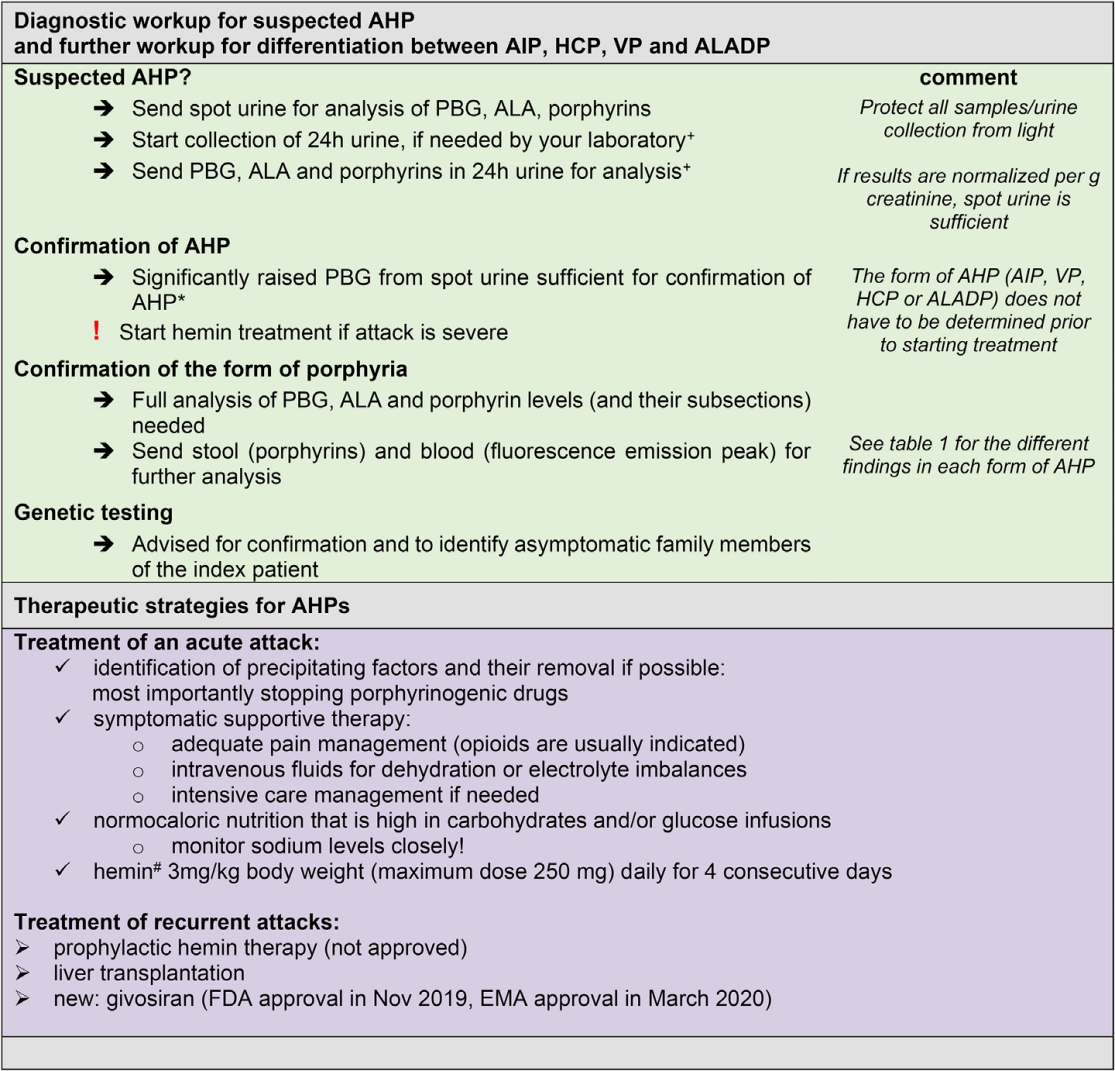
Diagnostic workup and therapeutic strategies in AHPs. +If laboratory results are available as metabolites normalized per gram creatinine, spot urine is sufficient. Otherwise, a 24 h urine collection is recommended. *ALADP, tyrosinemia type I and lead intoxication may be missed if only PBG is analyzed because they cause raised ALA and raised porphyrins only. ^#^Hemin is available in Europe as heme arginate (stable form of human hemin in a complex with arginine; NormosangÂ^®^, Orphan Europe, Puteaux, France) and in the United States and other countries as lyophilized human hemin (PanhematinÂ^®^, Recordati Rare Diseases, Lebanon, NJ, USA). AHP, acute hepatic porphyrias; AIP, acute intermittent porphyria; ALA, 5‐aminolaevulinic acid; HCP, hereditary coproporphyria; VP, variegate porphyria; PBG, porphobilinogen

Laboratory workup to exclude AHP should be done promptly in critically ill patients (Figure 3). It is inexpensive and can save the patient from receiving unnecessary procedures and porphyrinogenic drugs that worsen the attack (Anderson et al., 2005).

Diagnosis during an acute attack is based on detection of raised urinary metabolites. If AHP is suspected, spot urine should be sent immediately and if results can be given as metabolites normalized per gram creatinine, this is sufficient (Anderson et al., 2005). Some laboratories still require native 24 h urine collection for full analysis. Note that PBG and porphyrins are instable in sunlight. Urine sample and collection system should be protected from sunlight (e.g., wrapped in aluminum foil). Diagnosis can be established with raised PBG in spot urine. Some authors regard a two‐ to fourfold rise above the upper reference limit as sufficient for diagnosis (Kauppinen, 2005), whereas others require a more than ten‐fold increase (Puy et al., 2010). In VP and HCP, urine PBG may normalize within days and diagnosis may be missed with delayed sample collection (Stein et al., 2017).

Further workup should include urinary ALA, total porphyrins, and their subsections and fecal porphyrins. If only PBG is analyzed, ALADP, tyrosinemia type I, and lead intoxication will be missed as they only cause raised ALA and raised porphyrins. To distinguish between the porphyrias, additional measurement of erythrocyte PBGD‐activity and plasma fluorescence emission peak are helpful (overview of laboratory findings in Table 1, diagnostic workup Figure 3).

Genetic testing is advised for confirmation and to identify asymptomatic family members (Stein et al., 2017). Especially in VP and HCP, but also seldom in AIP, asymptomatic carriers may display normal urinary and fecal metabolite levels. Table 1 indicates the gene locus for each AHP. Most mutations are family‐specific and several different mutations have been described for each AHP (Anderson et al., 2005; Stein et al., 2017; Stolzel et al., 2019).

## 5 PRECIPITATING FACTORS FOR AN ACUTE ATTACK

Most precipitating factors increase demand for hepatic heme and directly or indirectly induce ALA synthase: endogenous hormones (particularly progesterone), exogenous hormones (oral contraceptives), alcohol, dieting/reduction in calories or carbohydrates, metabolic stress from infections/surgery, possibly psychological stress, and most importantly, many drugs can trigger an acute attack (Anderson et al., 2005; Stein et al., 2017; Windebank & Bonkovsky, 2005). Pregnancy is usually well tolerated, but induces attacks in some women (Anderson et al., 2005).

In each of our three cases we identified a combination of precipitating factors that always included porphyrinogenic drugs. It cannot be emphasized enough that, when suspecting porphyria, safe medication alternatives should be sought, unless an acutely life‐threatening situation needs to be handled. There is evidence that unsafe medications are the most important trigger for developing porphyric neuropathy (Windebank & Bonkovsky, 2005).

Two databases collect drug‐safety information for porphyrias: the nordic drug database “napos” (http://www.drugs‐porphyria.org) (Europe) and the database of the American Porphyria Foundation “apf” (http://www.porphyriafoundation.com/drug‐database) (USA). Classification is usually based on few reports and this may lead to contradictory information in some cases. A privately run website displays the information of both databases as an overview for each drug (http://porphyriadrugs.com). Table 4 gives an overview of the safety of drugs commonly used in neurology and psychiatry, but is not exhaustive (Albers & Fink, 2004; Anderson et al., 2005; Crimlisk, 1997; Windebank & Bonkovsky, 2005).

**TABLE 4:** List of safe and unsafe medications

UNSAFE MEDICATIONS	SAFE MEDICATIONS
Pain killers	
Metamizole/Dipyrone	ASS, Ibuprofen, Paracetamol
	Morphine, Hydromorphone
Antiemetics	
**Dimenhydrinate**	MCP
	Ondansetron
Antibiotics/antivirals	
Clindamycin	Azithromycin
**Erythromycin**, (Clarithromycin)	Carbapenems
(Flucloxacillin)	Cephalosporins
Isoniazid	Ciprofloxacin, Moxifloxacin
**Nitrofurantoin**	Doxycycline
**Rifampicin**	Gentamicin
**Sulfonamides**, **Trimethoprim**	Penicillins^a^
	Vancomycin
	Aciclovir, Ganciclovir, Valaciclovir
Antiepileptic therapy	
**Barbiturates (Phenobarbital)**	Gabapentin
Carbamazepin	Lacosamide
(Valproic acid)	Lamotrigine
Phenytoin	Levetiracetam
Primidone	Lorazepam, Midazolam
(Oxcarbazepine)	Magnesium
Topiramate	Pregabalin Propofol
Neuroleptics	
Melperone	Chlorpromazine
	Haloperidol
	Levomepromazin
Antidepressants	
(Imipramin)	Amitriptyline
	Duloxetine
	Mirtazapine
	SSRIs
Steroids	
(Dexamethasone)	Prednisolone
(Methylprednisolon)	Triamcinolone
Others	
Hormones	Atropine
Progesterone	Betablockers
	Catecholamines

*Note*: All drugs are classified after their safety in systemic use in a patient. The list is not comprehensive and the authors advise to check in the following databases for updated information on a drug before prescription to a patient with porphyria: http://www.drugs‐porphyria.org or http://www.porphyriafoundation.com/drug‐database or http://porphyriadrugs.com

Drugs in bold are regarded as unsafe and have reportedly triggered attacks. Drugs in parenthesis either have conflicting records in the two databases or are regarded as possibly unsafe or only probably safe.

^a^ Flucloxacillin is regarded as possibly unsafe.

## 6 HEME DEFICIENCY OR ALA‐INDUCED TOXICITY?

**FIGURE 4:**
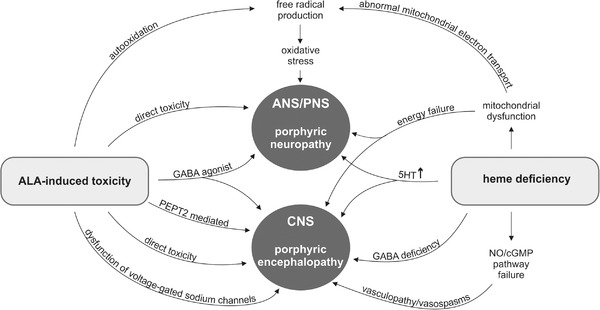
ALA‐induced toxicity or heme deficiency? Proposed overview of main hypotheses of pathophysiological mechanisms of neurotoxicity. For detailed explanation, please refer to main text. ALA, 5‐aminolaevulinic acid; ANS, autonomic nervous system; cGMP, cyclic guanosine monophosphate; CNS, central nervous system; GABA, gamma amino butyric acid; 5HT, serotonin; NO, nitric oxide; PEPT2, peptide transporter 2; PNS, peripheral nervous system

The exact pathophysiological mechanisms that lead to nerve cell dysfunction and ultimately cause the neuropathy and encephalopathy syndromes remain unclear. Mainly, two pathophysiology hypotheses are being discussed: critical heme deficiency or ALA‐induced toxicity. We propose an overview of these hypotheses and their supposed pathophysiological mechanisms of neurotoxicity (Figure 4). Heme is essential for the respiratory chain and several other enzymes. A critical lack of heme could cause mitochondrial dysfunction and lead to imbalance in several transmitter systems within the nervous system (Chacko et al., 2019; Demasi et al., 1996; Homedan et al., 2015; Lin et al., 2013; Thunell, 2000). Further, heme deficiency may lead to mitochondrial dysfunction, causing a general energy failure in the cell and causing abnormal mitochondrial electron transport that leads to the production of free radicals and causes oxidative stress (Demasi et al., 1996; Lin et al., 2013; Thunell, 2000). Measurements of cellular bioenergetics and mitochondrial respiration have recently shown evidence of mitochondrial dysfunction in patients with porphyria and in a mouse‐model of AIP (Chacko et al., 2019; Homedan et al., 2015). Heme deficiency might also lead to an impairment of tryptophan pyrollase, leading ultimately to increased generation of 5‐HT (serotonin or 5‐OH‐tryptamine). Several autonomic symptoms in acute porphyrias could be explained through activation of 5‐HT receptors (Lin et al., 2013; Thunell, 2000). At a low energy level in nerve cells, GABA production could be compromised leading to nerve cell dysfunction (Thunell, 2000). Restricted availability of heme could lead to impairment of the heme protein nitric oxide synthase and cause a lack of NO (nitric oxide) and thus lead to vasculopathy and even vasospasms (Takata et al., 2017; Thunell, 2000).

The hypothesis of ALA‐induced toxicity is based on the fact that the porphyrin precursor ALA is significantly elevated in all four acute hepatic porphyrias. ALA is toxic to neuronal and glial cells in culture and induced neurological symptoms in rats (Lin et al., 2013). However, these neurotoxic effects are only seen at concentrations much higher than ever found in patients (Emanuelli et al., 2000). On the other hand, low extracellular concentrations of ALA affected voltage‐gated sodium channels in a study on isolated rat hippocampal neurons (Wang et al., 2005). Furthermore, there is evidence that the CSF‐blood barrier, which is maintained by the choroid plexus, is transporter mediated (Ennis et al., 2003). In peptide transporter 2 (PEPT2)‐deficient mice, significantly higher concentrations of ALA in CSF after exposure were found than in wild‐type mice (Hu et al., 2007). PEPT2 has a high affinity for ALA and is found in the kidneys and in the choroid plexus. In the latter, it might determine the amount of toxic ALA that reaches the brain and therefore PEPT2 might be a secondary genetic modifier explaining the variable susceptibility to neuropsychiatric symptoms in patients (Hu et al., 2007; Stolzel et al., 2019). In addition, ALA may compete for GABA binding sites and act as a GABA agonist (Lin et al., 2013). Finally, ALA is known to form free radical species on auto‐oxidation, thus causing oxidative stress (Lin et al., 2013; Thunell, 2000).

One of the main arguments against the hypothesis of heme deficiency in nerve cells is the fact that liver transplant leads to clinical and biochemical remission in AIP patients and that recipients of a liver from AIP‐donors developed acute attacks and a porphyric neuropathy (Dowman et al., 2011; Seth et al., 2007; Singal et al., 2014; Soonawalla et al., 2004). On the other hand, ALA is significantly elevated in all four AHPs is and considered neurotoxic. However, asymptomatic high excreters exist and ALA levels do not always correlate with symptom severity or with recovery. Therefore, raised ALA levels seem to be a necessary but not a sufficient precondition for generating the neurovisceral symptoms in AHPs.

## 7 NEUROLOGICAL DIAGNOSTIC FINDINGS

### 7.1 Brain imaging

In the literature, 53 cases with PRES‐like lesions in brain MRI during acute porphyric attacks are reported (Aggarwal et al., 1994; Bhat et al., 2010; Bhuyan et al., 2014; Bicknell & Stewart, 2011; Black et al., 1995; Bonkovsky et al., 2008; Celik et al., 2002; Dagens & Gilhooley, 2016; Dahlgren et al., 2011; Divecha et al., 2016; Engelhardt et al., 2004; Garg et al., 1999; Gurses et al., 2008; Jaramillo‐Calle et al., 2019; Kang et al., 2010; King & Bragdon, 1991; Kuo et al., 2011; Kupferschmidt, 1995; Lakhotia et al., 2015; Lambie et al., 2018; Maramattom et al., 2005; Mullin et al., 2012; Mutyaba et al., 2011; New et al., 2016; Olivier et al., 2017; Park et al., 2014; Pichler et al., 2015; Sakashita et al., 2017; Sanz et al., 2016; Shen et al., 2008; Silveira et al., 2016; Soysal et al., 2008; Susa et al., 1999; Takata et al., 2017; Utz et al., 2001; Webb et al., 2016; Wessels et al., 2005; Yang et al., 2017; Yen et al., 2002; Yrjonen et al., 2008; Zhang et al., 2017; Zhao et al., 2014; Zheng et al., 2018). Described lesions are symmetrical or asymmetrical, ranging from bioccipital white matter lesions to gyriform cortical lesions, some show contrast enhancement (Aggarwal et al., 1994; Black et al., 1995; Maramattom et al., 2005; Susa et al., 1999; Zhao et al., 2014; Zheng et al., 2018) and most are in posterior circulation territories. Some were associated with mild‐to‐moderate (Black et al., 1995; Celik et al., 2002; King & Bragdon, 1991; Lakhotia et al., 2015; Lambie et al., 2018; Mullin et al., 2012; Soysal et al., 2008; Takata et al., 2017; Utz et al., 2001; Yang et al., 2017) up to severe hyponatremia (Sanz et al., 2016; Susa et al., 1999; Webb et al., 2016; Yrjonen et al., 2008; Zheng et al., 2018); others, with increased blood pressure (Bhuyan et al., 2014; Bonkovsky et al., 2008; Celik et al., 2002; Dahlgren et al., 2011; King & Bragdon, 1991; Kupferschmidt, 1995; Lakhotia et al., 2015; Olivier et al., 2017; Sakashita et al., 2017; Utz et al., 2001; Wessels et al., 2005; Yen et al., 2002; Yrjonen et al., 2008; Zhang et al., 2017; Zhao et al., 2014), though mostly not reaching levels of malignant hypertension (Pischik & Kauppinen, 2009). Because of cases with PRES‐like lesions with normal sodium levels and only slightly raised blood pressure, alternative theories of endothelial toxicity leading to vasogenic edema (Engelhardt et al., 2004; Fischer & Schmutzhard, 2017; Kupferschmidt, 1995; Pischik & Kauppinen, 2009; Takata et al., 2017) and reversible vasospasms have been discussed (Aggarwal et al., 1994; King & Bragdon, 1991; Kupferschmidt, 1995; Soysal et al., 2008; Susa et al., 1999). Six cases report confirmed cerebral vasospasms during an acute attack (Black et al., 1995; Maramattom et al., 2005; Mullin et al., 2012; Olivier et al., 2017; Takata et al., 2017; Webb et al., 2016), in four cases leading to infarctions possibly due to prolonged duration of vasospasms (Black et al., 1995; Mullin et al., 2012; Olivier et al., 2017; Takata et al., 2017). Additionally, a series of seven HCP patients reported cerebral hypoperfusion on SPECT during attacks that was partly reversible in follow‐up imaging and not detected on MRI (Valle et al., 2016).

However, two case reports detected signs of cerebral hyperperfusion associated with PRES‐like lesions, one in ASL MRI (Sakashita et al., 2017) and one through increased flow in duplex sonography (Utz et al., 2001). These controversial findings are in line with findings in PRES of other causes (Fischer & Schmutzhard, 2017), where cerebral hyper‐ as well as hypoperfusion have been detected. Possibly, these controversial findings reflect different phases of the same pathophysiological process.

More commonly, MRI is unremarkable in patients with acute porphyria (Kuo et al., 2011) or shows small, unspecific findings that resemble cerebral microangiopathy or inflammatory diseases (Bylesjo et al., 2004).

In summary, brain imaging in AHPS is as heterogeneous as the clinical presentation. Acute porphyria should be included in the list of differential diagnoses for patients with acute encephalopathy and PRES‐like MRI‐findings.

### 7.2 EEG

The most frequently reported EEG finding in patients with acute porphyric attacks is a diffuse slowing to theta and delta frequency ranges. (Aggarwal et al., 1994; Celik et al., 2002; Engelhardt et al., 2004; King & Bragdon, 1991; Winkler et al., 2005; Zhao et al., 2014; Zheng et al., 2018) This is an unspecific finding correlating clinically with signs of encephalopathy.

### 7.3 CSF findings

CSF results of patients with acute porphyric attacks and neurological symptoms are either normal (Pischik & Kauppinen, 2009) or reveal an albuminocytological dissociation (Bylesjo et al., 2004; Windebank & Bonkovsky, 2005). This highlights the diagnostic difficulty in discriminating GBS from acute porphyric neuropathy. There are rare reports of pleocytosis in CSF during acute attacks (Lyons, 1950).

Measurement of porphyrin precursors in CSF showed low detectable CSF levels of ALA and PBG during acute attacks in three VP patients (Percy & Shanley, 1977). In a severely affected infant with homozygous AIP, elevated CSF levels of PBG were found (Solis et al., 2004).

### 7.4 Nerve conduction studies/electromyography

An estimated 10%–40% of symptomatic patients develop a porphyric neuropathy (peripheral and autonomic neuropathy) (Albers & Fink, 2004; Simon & Herkes, 2011). There are no pathognomonic clinical findings and NCS findings are heterogeneous as well. Most cases report a predominantly axonal neuropathy with reduced motor amplitudes and normal or only slightly affected conduction velocities and distal latencies (Albers & Fink, 2004; Andersson et al., 2002; Celik et al., 2002; King & Bragdon, 1991; Kuo et al., 2011; Kupferschmidt, 1995; Lin et al., 2011; Simon & Herkes, 2011; Soysal et al., 2008; Wessels et al., 2005; Wu et al., 2015). However, signs of demyelination (temporal dispersion, conduction block, and prolonged F‐wave latencies) have been reported (Barohn et al., 1994; King et al., 2002; Pischik & Kauppinen, 2009; Wu et al., 2015; Younger & Tanji, 2015). Sensory nerve involvement is usually absent or mild (Albers & Fink, 2004; Andersson et al., 2002; Simon & Herkes, 2011). Needle electromyography showed fibrillations most prominently in proximal muscles within several weeks of onset, indicating denervation. Patients studied during the recovery phase showed evidence of ongoing reinnervation (Albers & Fink, 2004; Lin et al., 2011; Simon & Herkes, 2011). Most of these findings are reversible, but cases with chronic persistent neuropathy have been reported (Kuo et al., 2011; Lin et al., 2011; Wu et al., 2015).

### 7.5 Neuropathology findings

Histopathologic changes have been documented at all levels of the nervous system, with controversial findings. Autopsy findings are especially difficult to interpret because of additional changes caused by prolonged disease course and influence of terminal illness, such as sepsis (Simon & Herkes, 2011; Suarez et al., 1997; Windebank & Bonkovsky, 2005).

Findings from sural nerve biopsies demonstrate a loss of mainly large myelinated fibers, Wallerian‐like degeneration, and macrophagic activity (Barohn et al., 1994; Bonkovsky et al., 2008; Di Trapani et al., 1984; Tracy & Dyck, 2014), indicating a primarily axonal lesion. However, segmental demyelination (Anzil & Dozic, 1978) and remyelination (Younger & Tanji, 2015) have also been reported. Autopsy findings support a predominantly axonal damage affecting mostly the large myelinated fibers in multiple peripheral nerves (Cavanagh & Mellick, 1965; Gibson & Goldberg, 1956; Hierons, 1957; Suarez et al., 1997; Yamada et al., 1984). However, single cases with demyelination of the vagus nerve and of fibers of the sympathetic chain have been reported (Gibson & Goldberg, 1956). Involvement of small fibers has been demonstrated with reduced intra‐epidermal nerve fiber density in skin biopsies (Hsieh et al., 2007; Younger & Tanji, 2015).

Muscle biopsies obtained from autopsies demonstrate neurogenic changes (denervation atrophy as a sign of denervation with fiber type grouping as a sign of reinnervation) and no clear evidence of myopathic changes (Bonkovsky et al., 2008; Cavanagh & Mellick, 1965; Suarez et al., 1997; Yamada et al., 1984; Younger & Tanji, 2015).

In the spinal cord, autopsy cases showed chromatolysis and reduction in number of anterior horn cells (Cavanagh & Mellick, 1965; Hierons, 1957; Yamada et al., 1984) and degeneration and loss of ganglion cells in dorsal root ganglia (Yamada et al., 1984).

Finally, in the cerebrum and cerebellum, either no relevant changes on autopsy (Hierons, 1957; Suarez et al., 1997; Yamada et al., 1984), or small demyelinating lesions (Gibson & Goldberg, 1956), ischemic lesions, (Hierons, 1957; Suarez et al., 1997), and selective reduction of hypothalamic neurons (Suarez et al., 1997) have been reported. None of these findings are correlated with imaging studies and are therefore difficult to interpret.

## 8 TREATMENT

Treatment of acute attacks consists of four major points (Figure 3): First, identification of precipitating factors and their removal if possible. Porphyrinogenic drugs should be stopped. Second, symptomatic supportive therapy, including adequate pain management (opioids are usually indicated), correction of electrolyte imbalances, and intensive care management, if needed. Third, normocaloric nutrition high in carbohydrates and/or glucose infusions. Sodium levels need to be monitored closely.

Finally, severely ill patients should receive hemin (3 mg/kg body weight [up to a maximum of 250 mg] daily for four consecutive days) (Stein et al., 2017; Stolzel et al., 2019). Hemin is indicated in patients with AHP and any neurological symptoms, in those requiring intensive care and in some cases, for pain management. Heme arginate can be reconstituted in 100 ml normal saline or 100 ml 20% human serum albumin and should be administered over a central intravenous line to avoid vein irritation (Bonkovsky et al., 1991; Stein et al., 2017). Thrombophlebitis is the most common side‐effect and rarely vein obliteration occurs. Hemin acts as a transcription factor that reduces expression of the rate‐limiting hepatic enzyme ALAS1. This leads to down‐regulation of the entire pathway and reduction of toxic metabolites. For desperate cases with severe recurrent attacks, liver transplant has been performed leading to clinical and biochemical remission (Seth et al., 2007; Singal et al., 2014; Soonawalla et al., 2004; Stolzel et al., 2019).

Antiepileptic therapy can be done with Gabapentin, Lacosamide, Lamotrigine, Levetiracetam, Lorazepam, Midazolam, and Pregabalin. Addition of magnesium and propofol has been successful (Bhatia et al., 2008; Dawit et al., 2019; Engelhardt et al., 2004; Stolzel et al., 2019; Zaatreh, 2005). Unsafe antiepileptics that can worsen AHP should be strictly avoided, especially barbiturates, but also valproic acid and carbamazepine. For the full list of safe and unsafe medications, please refer to Table 4. Neuropathic pain is a common problem during acute attacks, but also during recovery. Pain management can be done with ASS, Ibuprofen, Paracetamol, opioids and with gabapentin or pregabalin.

In November 2019, the FDA, and in March 2020, the EMA approved a new therapeutic for patients with recurrent attacks: givosiran (Alnylam Pharmaceuticals, Cambridge, MA, USA) is a small interfering RNA (siRNA) that neutralizes excess ALAS1 mRNA in hepatocytes (Sardh et al., 2019; Stolzel et al., 2019). Recently published results of the ENVISION‐trial (NCT03338816), a phase III, multicenter, placebo‐controlled, randomized controlled trial, showed reduction of urinary ALA levels and of the number of acute attacks (Balwani et al., 2020). The siRNA is injected subcutaneously and has shown an acceptable safety profile so far (Balwani et al., 2020; de Paula Brandao et al., 2020; Sardh et al., 2019; Stolzel et al., 2019). It is not approved for treatment of acute attacks and to date, there is no data on its effect on residual neurological symptoms.

## 9 CONCLUSIONS AND FUTURE DIRECTIONS

Acute porphyrias can cause severe neurological symptoms that involve all levels of the nervous system. Due to their relative rarity and their chameleon‐like presentation, delayed diagnosis and misdiagnosis are common and can lead to severe sequelae and fatal outcome. In our opinion, acute porphyrias should be considered early and parallel to other differential diagnoses in the diagnostic workup of severely ill neurological patients who display an encephalopathic or GBS‐like syndrome. Red flags are neurological symptoms starting after severe (abdominal) pain, in association with reddish urine, hyponatremia, photodermatitis or known precipitating factors (alcohol, dieting, menstrual cycle, certain drugs), and presence of both encephalopathy and axonal neuropathy. If detected promptly and treated appropriately, prognosis of the acute porphyric attack is good and complete neurological recovery is possible.

Future research should be directed at elucidating the exact pathophysiological mechanisms causing the severe neuropathy and encephalopathy syndromes. Systematic data on the extent, quality, and duration of acute and residual neurological symptoms in these patients is lacking. Finally, future studies should investigate whether causal therapy, for example, with givosiran, can prevent neurological consequences and positively influence residual symptoms.

## REVIEW CRITERIA

We searched PubMed for articles in all year ranges with multiple combinations of search terms, including “porphyria,” “acute porphyria,” “acute intermittent porphyria,” "variegate porphyria,” “hereditary coproporphyria,” “neurological,” “neurology,” “MRI,” “imaging,” “PRES,” “neuropathology,” “neuropathy,” and “encephalopathy. There were no language exclusions and articles chosen were based on relevance to the topics covered in this review.

## AUTHOR CONTRIBUTIONS

Lea M. Gerischer and Andreas Meisel initially defined the scope of the review and designed the figures. All authors contributed to the writing and revision of all aspects of the manuscript.

## CONFLICT OF INTEREST

Lea M. Gerischer, Franziska Scheibe, Astrid Nümann, and Martin Köhnlein report no disclosures relevant to the current manuscript. Andreas Meisel received funding from the German Research Foundation (TRR167), Einstein Foundation (A‐2017‐406), Leducq Foundation (19CVD01), and the German Federal Ministry of Education and Research (02K16C220). Ulrich Stölzel has received speaking fees and travel grants from Alnylam.

### PEER REVIEW

The peer review history for this article is available at https://publons.com/publon/10.1002/brb3.2389

## Supporting information

Supplemental Tables
