# Supplementary material for: Acute porphyrias – A neurological perspective
Source: Brain Behav. 2021 Oct 17;11(11):e2389. doi: 10.1002/brb3.2389 (PMC8613433; doi:10.1002/brb3.2389)
Supplement: Supplementary file 1 — Supplemental Tables [file BRB3-11-e2389-s001.docx]

**Supplemental figures and tables**

for manuscript entitled

**Acute porphyrias – a neurological perspective**

Lea M Gerischer, MD^1,2^, Franziska Scheibe, MD^1,2^, Astrid Nümann, MD^1^, Martin Köhnlein, MD^1^, Prof Ulrich Stölzel, MD^3^, Prof Andreas Meisel, MD^1,2^

**Supplemental table I**

| **Supplemental table I**  **Laboratory results case 1 – acute intermittent porphyria**  Porphyrins and precursors during acute attack and after hemin therapy | | | | | | | |
| --- | --- | --- | --- | --- | --- | --- | --- |
| **day of ICU treatment** | **PBG** in  mg/d | **ALA**  in mg/d | **Total Porphyrins**  in µg/d | **Copro I**  in µg/d | **Copro III** in µg/d | **Uroporphyrins**  in µg/d | **Porphyrins in feces**  in µg/g |
| d 64 | 126 | 16.8 | 30195 | 116 | 562 | 29038 | - |
| **hemin therapy** (day 86-90) | | | | | | | |
| d 90 | 76 | 13.3 | 1289 | 56 | 352 | 708 | - |
| d 120 | 67 | 15.1 | 997 | 28 | 468 | 392 | 15.5 |
| d 135 | 79 | 22.2 | 1771 | 48 | 563 | 1005 | - |
| d 142 | 84 | 21.7 | 1774 | 35 | 342 | 1209 | - |
| normal values | < 1.7 mg/d | < 6.0 mg/d | < 150 µg/d | < 25 µg/d | < 75 µg/d | < 25 µ/d | < 34µg/g |
| Abbreviations: ALA: 5-aminolaevulinic acid; d: day; ICU: intensive care unit; Copro: Coproporphyrin; PBG: porphobilinogen. | | | | | | | |

**Supplemental table II**

| **Supplemental table II**  **Laboratory results case 2 – variegate porphyria**  Porphyrins and precursors in urine and feces during acute attack with severe neurological symptoms and after hemin therapy  and over the course of three years | | | | | | | | | | | |
| --- | --- | --- | --- | --- | --- | --- | --- | --- | --- | --- | --- |
|  | **PBG** in  mg/d | **PBG** in  mg/l | **ALA**  in mg/d | **ALA**  in mg/l | **Total Porphyrins**  in µg/d | **Total porphyrins**  in µg/l | **Uroporphyrins**  in µg/d | **Copro III**  in µg/d | **Copro III**  in µg/l | **Porphyrins in feces**  in µg/g |  |
| Acute attack (abdominal pain + neurological symptoms)^a^ | 79.2 | 132.0 | 57.6 | 96.0 | 1723 | 2873 | 857 | 602 | 1003 | - |  |
| Acute attack, after hemin therapy | 0.6 |  | 2.5 |  | 223 |  | 28 |  |  | 608 |  |
| Remission  4 months later^b^ | - | 1.3 | - | 4.1 | - | 271 |  | - | 232 | - |  |
| Remission  8 months later^b^ | - | 0.8 | - | 2.2 | - | 79 |  | - | 58 | - |  |
| Acute attack  (abdominal pain only)  3 years later^b^ | - | 6.5 | - | 14.0 |  | 1075 |  |  | 1008 | - |  |
| *normal values*  *(24h urine)* | *< 1.7 mg/d* | *<1.7mg/l* | *< 6.0 mg/d* | *<6.0mg/l* | *< 150 µg/d* | *-* |  | *< 75 µg/d* | *-* | < 85µg/g* |  |
| Abbreviations: PBG: Porphobilinogen, ALA: 5-aminolaevulinic acid; Copro: Coproporphyrin. ^a^ 24h-urine collection; ^b^ spot urine sample; * the different normal ranges for porphyrins in feces for case 1 and case 2 and 3 result from different laboratory facilities where the analyses have been performed. | | | | | | | | | | | |

**Supplemental figure I**. **MRI case 3**.

**
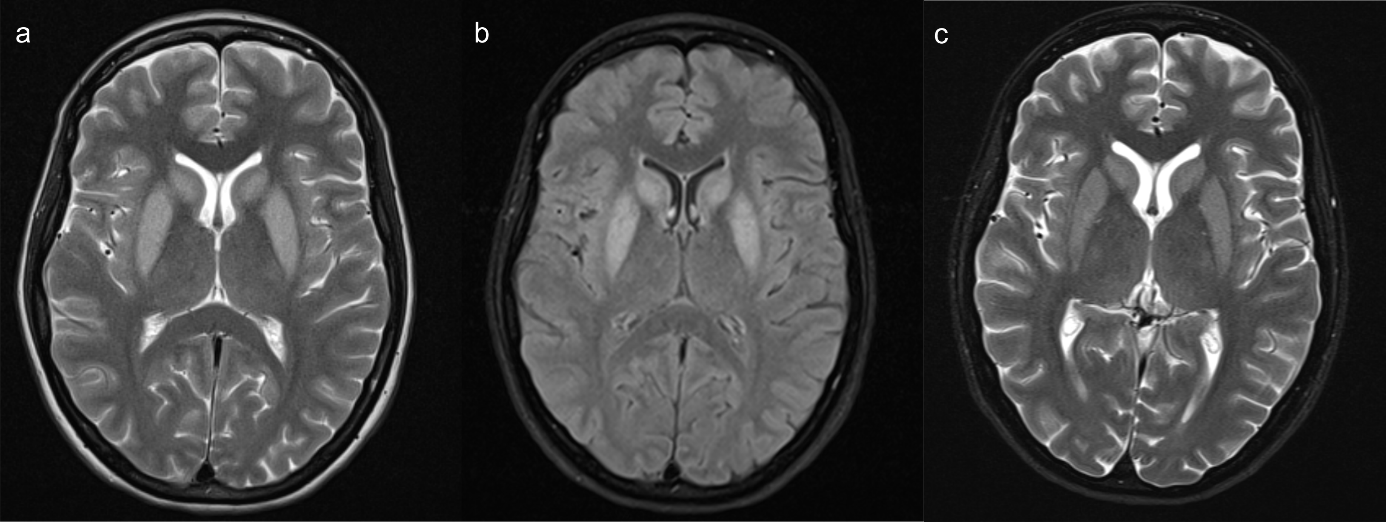
**

**Supplemental figure I**. **MRI case 3**. a) T2 and b) FLAIR of initial MRI with symmetrical hyperintensities bilaterally in the basal ganglia c) T2 of follow-up MRI with resolution of lesions.

**Supplemental table III**

| **Supplemental table III**  **Laboratory results case 3 – acute intermittent porphyria**  Porphyrins and precursors in urine and feces during acute attack and over the course of 1 year | | | | | | |
| --- | --- | --- | --- | --- | --- | --- |
|  | **PBG**  in µmol/g Crea | **ALA**  in µmol/g Crea | **Total Porphyrins**  in µg/g Crea | **Coproporphyrins**  in µg/d | **Uroporphyrins**  in µg/d | **Porphyrins in feces**  in µg/g |
| **Acute attack** | 514 | 564 | 3539 | 513 | 1430 | 114.6 |
| *Months after acute attack* |  |  |  |  |  |  |
| 5 | 143 | 99 | 1643 | 385 | 79 | - |
| 12 | 200 | 175 | 2505 | 1304 | 860 | - |
| normal values | <8 µmol/g Crea | <25 µmol/g Crea | < 174 µg/g Crea | < 100 µg/d | <27 µg/d | < 85µg/g* |
| Abbreviations: PBG: Porphobilinogen, ALA: 5-aminolaevulinic acid; Crea: Creatinine excretion in spot urine.  * the different normal ranges for porphyrins in feces for case 1 and case 3 result from different laboratory facilities where the analyses have been performed.  *Comment: values in this table are displayed as µmol/g Creatinine because spot urine has been analyzed for the follow-up examinations at month 5 and 12 after the acute attack. Normal ranges are different than from our in-house laboratory in supplemental table I and II.* | | | | | | |
